# A real-world experience of active surveillance in Bethesda IV thyroid nodules

**DOI:** 10.1007/s12020-026-04678-5

**Published:** 2026-07-13

**Authors:** Juan José Santivañez, Carlos Andrés García-Lozano, Carlos Betancourt, Juan Guillermo Sánchez, Laura Viviana Mendieta, David Figueroa-Bohórquez, John Diaz, Esteban Moreno-Castrillón, Alvaro Sanabria

**Affiliations:** 1 Head and Neck Surgery Section, Department of Surgery, Universidad de Antioquia, Medellin, Colombia; 2 Department of Surgery, Hospital Alma Mater de Antioquia, Medellin, Colombia; 3 Department of Surgery, Hospital Universitario San Vicente de Paul, Medellín, Colombia; 4 CEXCA. Centro de Enfermedades en Cirugia de Cabeza y Cuello, Medellín, Colombia; 5 Clínica CES, Medellín, Colombia; 6 Emergency department, Hospital Alma Mater de Antioquia. Medellin, Medellin, Colombia; 7 Cancer Unit. Clinica SOMER. Rionegro, Antioquia Rionegro, Colombia

**Keywords:** Thyroid nodule, Active surveillance, Lobectomy, Thyroidectomy, Growth, Follicular neoplasm

## Abstract

**Purpose:**

Management of Bethesda IV thyroid nodules is challenging due to their indeterminate nature and potential overtreatment. The aim was to evaluate the long-term outcomes of an active surveillance (AS) strategy for Bethesda IV nodules using clinical and ultrasound-based selection criteria in a middle-income setting without access to molecular testing.

**Methods:**

Retrospective analysis of a multicenter observational cohort. Patients were enrolled between January 2016 and December 2024. Adults with cytologically confirmed Bethesda IV thyroid nodules < 4 cm, TI-RADS 2–4, and no high-risk clinical or radiologic features were offered AS. Patients with prior head and neck cancer, prior radiation, or suspicious lymphadenopathy were excluded. AS with structured clinical and ultrasound follow-up. Surgery was considered for significant growth (≥ 3 mm), new suspicious features, or by clinical/patient decision.

**Results:**

A total of 184 patients (88.6% women, 52.7% <55 years) were included. At 48 months, 87.6% of nodules remained growth-free and 89.0% had not required surgery. Overall, 36 nodules (19.6%) showed growth; 26 patients (14.1%) underwent surgery. The malignancy rate was 7% for the full cohort and 46% among operated patients. Most malignant tumors were low-risk papillary carcinomas. No variable significantly predicted growth, but initial nodule size was associated with surgery (HR 1.06; 95% CI, 1.00–1.12; *P* = 0.04).

**Conclusions:**

In this cohort, AS for Bethesda IV nodules demonstrated high stability, low malignancy rates, and low surgical conversion, supporting its feasibility in selected patients. In resource-limited settings, AS guided by clinical and ultrasound criteria may safely reduce unnecessary surgeries.

**Supplementary Information:**

The online version contains supplementary material available at 10.1007/s12020-026-04678-5.

## Introduction

Thyroid cancer is the most common endocrine cancer, and its incidence has steadily increased in recent decades, due primarily to the indiscriminate use of diagnostic methods or the detection of indolent lesions in tests for other diseases [1, 2]. This increase has resulted in an overdiagnosis of low-risk carcinomas, with no significant effect on mortality, encouraging a reconsideration of more conservative treatments [3]. 

Thyroid nodules with indeterminate cytology, particularly those categorized as Bethesda IV (follicular neoplasm), pose a diagnostic challenge. Histological confirmation of capsular and vascular invasion is necessary for a conclusive diagnosis, supporting the conventional use of diagnostic lobectomy [4, 5] However, a large proportion of these nodules (65–70%) are benign, and when malignant, they usually are follicular subtypes of papillary carcinoma (PTC) or noninvasive follicular neoplasia with papillary-like nuclear characteristics (NIFTP), with indolent clinical behavior [6]. 

Molecular testing has been advocated as a strategy for improving patient selection and reducing avoidable surgeries [7–9]. However, its widespread use in middle-income countries is restricted by high cost and availability, and their incremental advantage over adequate clinical-ultrasound stratification may not always justify the investment. [10,11]

Several studies indicate that ultrasonography (US) features according to the TI-RADS scores may be used to stratify malignancy risk and guide the initial treatment of thyroid nodules [4, 10, 11]. The growth of Bethesda IV nodules with low- or intermediate-risk US features is low, allowing for alternative approaches like active surveillance (AS). This approach, which has been used for thyroid nodules classified as Bethesda V-VI [12–14], has been found to be safe. AS in Bethesda IV nodules is not intended to predict or exclude malignancy, but rather to identify the optimal timing for intervention while avoiding overtreatment. Nodule stability provides clinically meaningful information supporting the safety of deferring surgery or molecular tests without compromising oncological outcomes, analogous to the rationale for AS in low-risk papillary carcinoma cohorts.

Although specific data for Bethesda IV nodules is still scarce, some research indicates that the clinical course is typically indolent [15–17]. However, studies with larger sample sizes and longer follow-up periods are required.

We previously reported our findings in a cohort of 52 patients with a median follow-up of 12 months and nodule stability of more than 80% [15]. Accordingly, we believe that AS, when combined with well-defined clinical and US criteria, might provide an acceptable alternative for patients with Bethesda IV nodules, avoiding surgeries and optimizing healthcare costs. The current study aims to update the previously reported cohort with a significantly greater sample size, longer follow-up period, and a broader analysis of outcomes.

## Materials and methods

The study was approved by the institutional Research Ethics Committee, and all procedures adhered to the principles outlined in the Declaration of Helsinki. Patient confidentiality was strictly maintained. Informed consent was not required for this study due to its design. The option of AS for Bethesda IV nodules had been previously evaluated in our institution in a separate study [15], which demonstrated its feasibility and safety in selected patients. Based on those findings, AS became an accepted alternative. Although this approach deviates from the traditional standard of immediate surgery, it has also been explored in other published series, further supporting its clinical justification [16, 17]. Therefore, patients were not required to provide specific informed consent beyond routine clinical documentation.

### Study design and population

Between January 1, 2016, and December 31, 2024, four institutions in Medellín, Colombia, recruited patients with an US diagnosis of a thyroid nodule and Bethesda IV cytology.

At the surgeon’s discretion, patients over 18 years of age with thyroid nodules smaller than 4 cm, TI-RADS 2–4 US classification and Bethesda IV cytologically confirmed were included in a AS program with periodic clinical and US follow-up. At the time of patient recruitment, the widely accepted management approach in these centers was diagnostic lobectomy for nodules < 4 cm, according to the 2015 ATA guidelines [4]. Following an explanation of the risks, advantages, and alternatives, the patient agreed to monitoring. Patients having a history of other head and neck cancer, radiation treatment to the head and neck, TI-RADS 5 and clinical or imaging indications of high-risk malignancies (extrathyroidal extension, suspicious lymph nodes, or distant metastases) at the time of first evaluation were excluded. The presence of comorbidities was not an explicit reason for choosing AS. Routine serum calcitonin was not measured.

### Diagnostic procedures

Patients were initially evaluated by their primary physician, internist, or endocrinologist, who controlled their clinical care, and then sent to each institution’s Head & Neck Surgery Department to decide whether surgical treatment was required. All H&N surgeons were trained in AS for Bethesda V-VI nodules and adhered to the previously described protocol [18]. 

Radiologists performed thyroid US for all patients using ACR TI-RADS criteria [10]. Cytological confirmation of Bethesda IV was done using fine needle aspiration biopsy (FNAB) and assessed by pathologists in accordance with the Bethesda System 2017 criteria [5]. Radiological and pathological evaluations were not centralized, therefore the data utilized for decision-making are consistent with reports seen in everyday clinical practice. No molecular tests were performed on any patient.

### Follow-up and outcomes

Neck US were done at 6-month intervals during the first 18 months and annually thereafter. Examinations were performed by different radiologists across participating centers, without restriction to specific operators or centralized image review. If the patients received levothyroxine at the first clinical evaluation, the treatment was continued as prescribed by the treating physician. The primary outcome of the study was defined as significant nodule growth (an increase of ≥ 3 mm in any single dimension) relative to baseline US measurements. Secondary outcomes included the need for surgery and histologically confirmed malignancy. Percentage changes in volume were not used to define growth, based on prior evidence suggesting that volumetric assessments may overestimate growth, particularly in small thyroid nodules [19]. Instead, growth criteria were based exclusively on linear measurements, which are more reproducible in real-world settings. As a post-hoc analysis, an alternative growth criterion of ≥ 20% increase in diameter and an absolute increase of at least 2 mm from baseline was also reported.

Surgery was considered in cases of growth > 3 mm, or when new sonographic features were identified that raised the TI-RADS categorization. These assessments were performed at each clinical encounter.

The decision to proceed with surgery could also be influenced by patient preference or the treating surgeon’s clinical judgment, even in the absence of objective growth. All surgical indications and decisions were documented. Outcome final evaluation was based on the patient’s status at the last available clinical visit.

Due to access constraints within the national healthcare system, the ongoing surveillance of clinically stable patients is often conducted by internists or endocrinologists. To ensure comprehensive outcome assessment, an active search through electronic medical records and insurance databases was conducted to identify follow-up visits not only with H&N surgeons but also with other healthcare providers. We consider that this multidisciplinary follow-up provides valuable information regarding nodule stability and the absence of surgical intervention.

If the patient was considered a surgical candidate, the extent of the surgery was decided by their treating surgeon. Patients with solitary, unilateral nodules < 4 cm were often considered candidates for lobectomy, whereas the remainder were considered candidates for total thyroidectomy. The surgeon decided to expand a lobectomy to a total thyroidectomy and include a neck dissection (CND), either preoperatively or intraoperatively, based on the discovery of a nodule with extrathyroidal extension or macroscopically suspicious lymph nodes. Our group does not routinely perform prophylactic CND and only reserves it in the context of cN1 patients [20]. In these patients, the final pathology results and the need for RAI were recorded into the database.

### Statistical analysis

Continuous variables were described as mean ± standard deviation or median. Categorical variables were presented as absolute and relative frequencies. Comparisons of continuous variables were performed using ANOVA, while categorical variables were analyzed with the chi-square test. Time-to-event outcomes were evaluated using Kaplan-Meier graph, with time calculated from the date of the first fine-needle aspiration biopsy (FNAB) to the last follow-up with any healthcare provider. Patients who did not experience the event of interest by the end of the study period were considered censored at their last recorded clinical contact. The log-rank test was used to assess differences in survival curves. We also conducted a post hoc multivariable analysis using Cox proportional hazards regression with time to growth and time to surgery as the dependent variables using age, sex, initial nodule size, and TI-RADS category as covariates. A p-value < 0.05 considered statistically significant. All analyses were performed using Stata software (version 16). Sankey diagrams to depict patient trajectories were generated using SankeyMATIC (http://sankeymatic.com/).

## Results

The study included 184 patients Bethesda IV thyroid nodules. Table 1 shows the patient’s clinical characteristics. Most patients (88.6%) were women, < 55 years (52.7%), had nodules < 2 cm (75%), had multiple nodules (76.6%), and were classified TI-RADS 4 (57.1%). 71.7% of patients had a follow-up larger than 24 months.

**Table 1:** Characteristics of patients

Variable	Value
**Demographics**	
**Age**,** mean ± SD (range)**	53.8 ± 14.5 years (13–88)
**Age ≥ 55 y**	87(47.3%)
**Female sex**,** n (%)**	163 (88.6%)
**Initial Nodule Size (mm)**	
Mean ± SD (range)	16.2 ± 6.9 mm (5–40), median 15 mm
0–9 mm, n (%)	25 (13.6%)
10–19 mm, n (%)	113 (61.5%)
20–40 mm, n (%)	46 (24.9%)
**Nodule Location**	
Right lobe, n (%)	105 (57.1%)
Isthmus, n (%)	46 (25.0%)
Left lobe, n (%)	33 (17.9%)
**Multinodularity**	141 (76.6%)
**TI-RADS Category**	
TI-RADS 2, n (%)	10 (5.4%)
TI-RADS 3, n (%)	69 (37.5%)
TI-RADS 4, n (%)	105 (57.1%)
**Follow-Up Duration**	
**Time in months (mean ± SD (range)**,** median)**	46.3 ± 30.0 (0.7–117.4), median 42.6
**1–11.9 m** **12–23.9 m** **> 24 m**	27 (14.7%) 25 (13.6%) 132 (71.4%)

### Nodule behavior

During follow-up, 36 patients (19.6%) showed a growth > 3 mm, whereas 124 (67.4%) remained stable. In addition, 43 patients (23.4%) showed a growth > 20% of the initial diameter. In the growth subgroup, 17 patients (47.2%) underwent surgery at 31.9 ± 20.4 months (2.3–67.9), with a histological diagnosis of benignity in 8 (47%) and malignancy in 9 (53%). Six patients had a classic subtype of PTC, two had minimally invasive FC, and one had a low-risk medullary carcinoma. Supplementary Table 1. The remaining 19 patients (52.7%) remained on AS due to surgeon or patient decision, with no evidence of further growth.

Among the 124 stable nodules, 9 (7.2%) were surgically treated due to the surgeon or the patient decision at 36.1 ± 20 months (14.7–77.3); 5 (55.5%) were benign, 3 (33.3%) were malignant, and one case (11.1%) did not have histological data. Two of the malignant tumors were classic PTC and one was a minimally invasive FC. The remaining 115 patients (92.7%) remained under surveillance. Neither of the 24 nodules that decreased in size required surgery during the follow-up period. None of the patients diagnosed with malignancy developed distant metastases during the follow-up period.

**Fig. 1:**
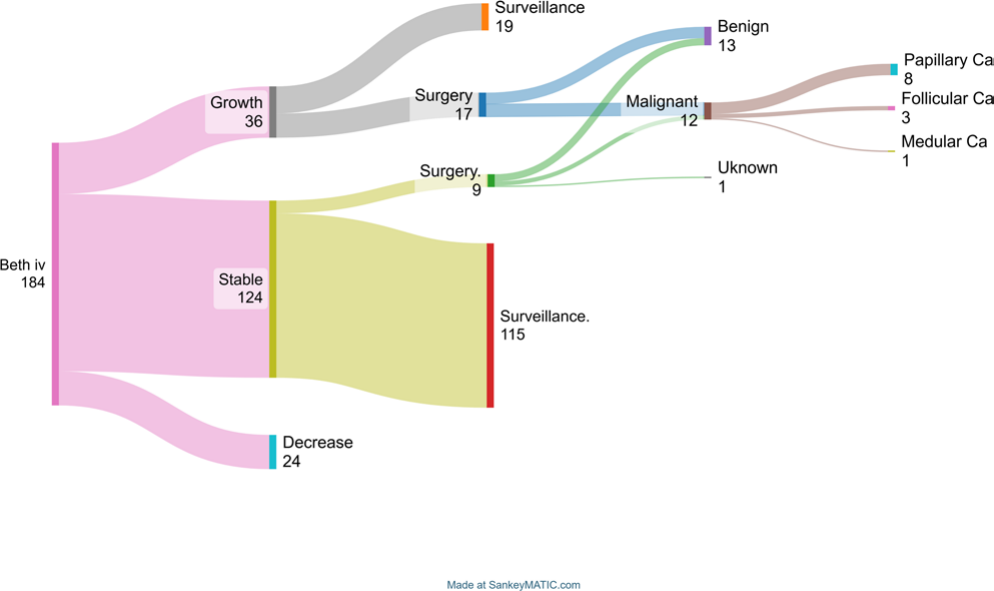
Evolution of thyroid nodules with Bethesda IV cytology under active surveillance, showing the distribution according to growth, stability or decrease in size, and the histopathological results in cases undergoing surgery

The outcomes for each group are shown in Fig. 1. The overall risk of malignancy in patients with growth > 3 mm and those operated were 9/36 (25%) and 12/26 (46.1%), respectively. Supplementary Fig. 1 shows the individual behavior of the nodules based on their initial size.

**Fig. 2:**
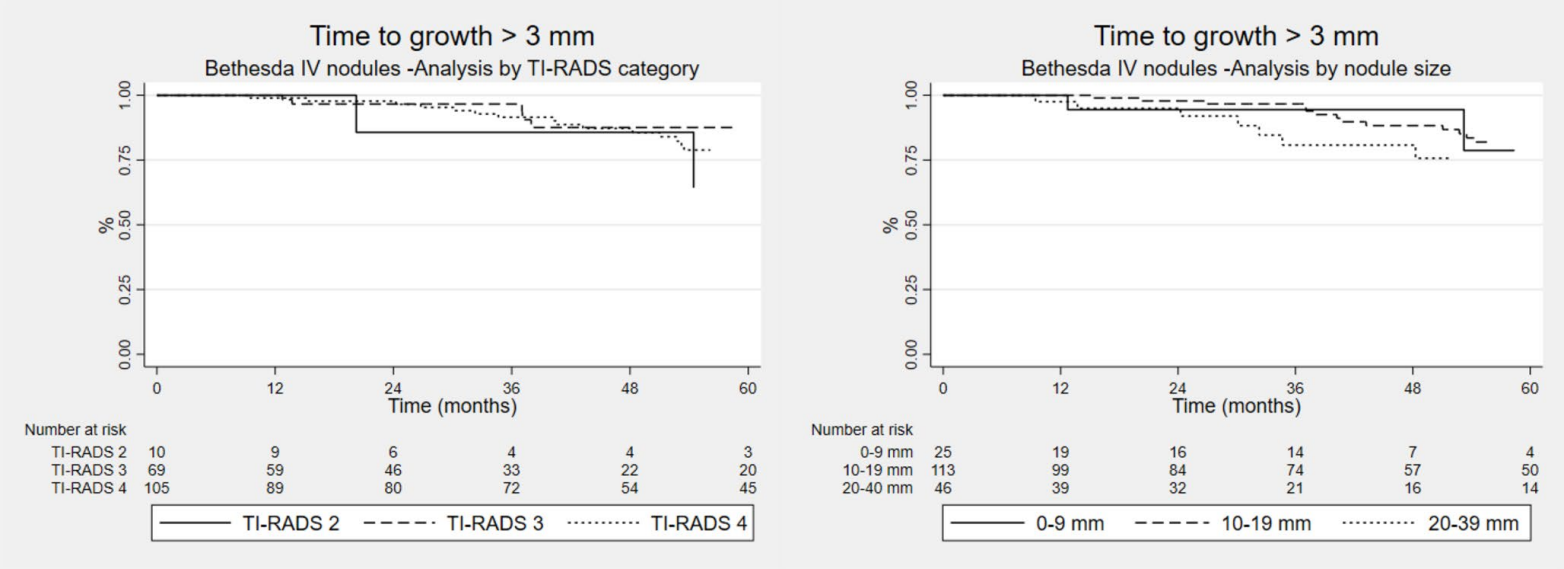
Stability of Bethesda IV nodules. (**A**) Time to growth > 3 mm by TI-RADS classification. (**B**) Time to growth > 3 mm by nodule size

The nodule stability until significant growth at 48 months was 87.6% (95%CI:79.6–91.3). No statistically significant differences in nodule stability were observed when stratified by TI-RADS category. Figure 2a At 48 months, 89.0% of patients were free of surgery (95%CI:81.7–93.5). Figure 2b.

The analysis of the growth criterion > 20% in diameter according to the TI-RADS classification and nodule size is shown in Supplementary Table 3.

### Analysis by TI-RADS classification

**Fig. 3:**
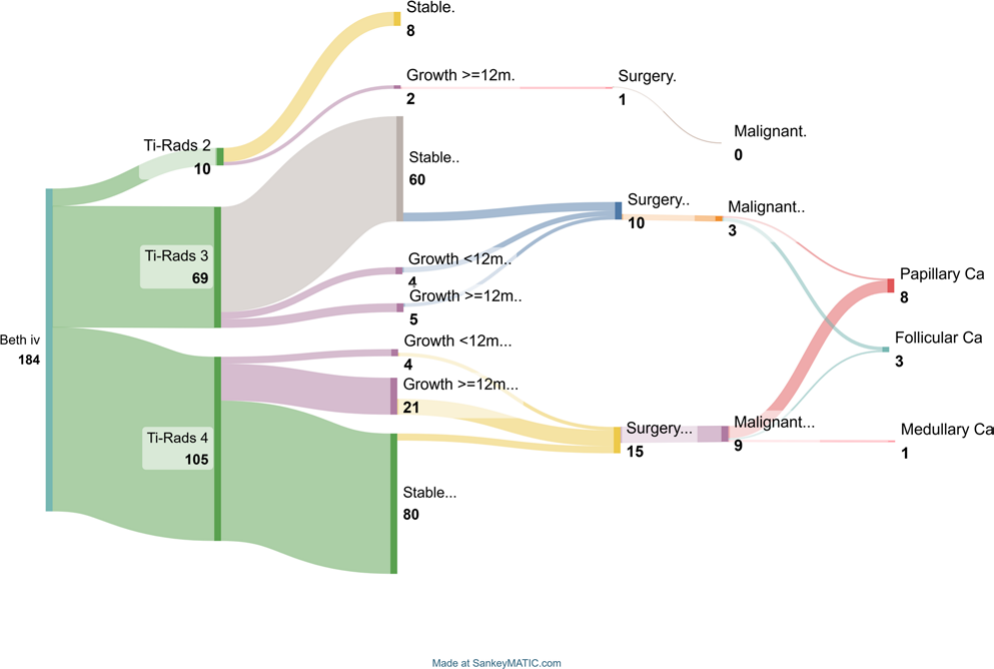
Evolution of thyroid nodules with Bethesda IV cytology under AS, showing the distribution according to TI-RADS category, and the histopathological results in cases undergoing surgery

Most nodules were categorized as TI-RADS 4 (*n* = 105, 57.1%). Overall, 148 patients (80.3%) demonstrated stability during follow-up, including 76.2% (80/105) of those with TI-RADS 4 nodules, 87.0% (60/69) with TI-RADS 3, and 80.0% (8/10) with TI-RADS 2. Surgical intervention and malignancy rates stratified by TI-RADS category are detailed in Table 2; Fig. 3. Although the risk of malignancy increased with higher TI-RADS categories, no statistically significant differences in clinical outcomes were observed between groups.

**Table 2:** Characteristics of nodules according to TI-RADS classification

*n*	TI-RADS 2	TI-RADS 3	TI-RADS 4	Total	*P* value
	10	69	105	184	
**Size at initial evaluation (mm)**					
Mean size +- SD (range)	15.6 ± 6.9 (9–29)	17.6 ± 7.3 (5–37)	15.3 ± 6.5 (5–40)		0.09
0–9 mm	1	7	17	25	0.09
10–19 mm	7	37	69	113	
20–40 mm	2	25	18	45	
**Size change (mm)** **(**Mean +- SD (range)	-0.4 ± 4 (-9 to 7)	-0.5 ± 5.4 (-28 to 13)	1.3 ± 5.2 (-13 to 22)		
**Time of follow-up (**Mean +- SD)	39.8 ± 30.9	41.3 ± 28.7	50.3 ± 30.5		0.11
**> 24 m (%)**	6 (60%)	46 (66.7%)	80 (76.2%)	132 (71.7%)	0.18
**Growth > 3 mm (n**,** %)**	2 (20,0%)	9 (13.0%)	25 (23,8%)	36 (19,6%)	0.21
Growth > 20%mm AND > 2 mm initial diameter*	2 (20%)	13 (18.8%)	28 (26.7%)	43 (23.4%)	0.47
**Underwent surgery (n**,** %)**	1 (10%)	10 (15%)	15 (14%)	26 (14.3%)	0.92
**Malignancy in operated**	0	3(30%)	9 (60%)	12 (46%)	0.27
**Malignancy in growth> 3 mm**	0	3(33%)	9(36%)	12(33%)	0.27
**Malignancy by group**	0	3 (4%)	9 (9%)	12 (7%)	0.37
**Nodules without growth > 3 mm at 48 m**	86.6%	89.5%	88.2%	87.6%	0.75
**Nodules without surgery at 48 m**	86.6%	89.6%	91.5%	89.0%	0.67

* All cases meeting the ≥ 20% diameter growth criterion also fulfilled an absolute diameter increase of ≥ 2 mm from baseline

### Analysis by size

Based on nodule size, most nodules were between 10 and 19 mm (*n* = 113, 61.4%). Overall, 148 patients (80.4%) demonstrated stability during follow-up, including 88% (22/25) of those with 0–9 mm nodules, 79.7% (90/113) with 10–19 mm, and 78.3% (36/46) with 20–40 mm. Surgical intervention and malignancy rates stratified by size are detailed in Supplementary Tables 1 and supplementary Fig. 2. Although no statistically significant differences in outcomes were observed based on initial nodule size, a trend was noted whereby the proportion of nodules remaining stable and managed without surgery decreased as size increased.

In the multivariable regression analysis, none of the assessed variables were significantly associated with nodule growth ≥ 3 mm. However, when surgery was used as the endpoint, initial nodule size emerged as a significant predictor (HR 1.06; 95%CI 1.00–1.12; *p* = 0.04). Other variables did not reach statistical significance in predicting surgery.

## Discussion

Bethesda IV thyroid nodules pose a diagnostic challenge, as distinguishing between follicular adenoma and carcinoma requires histological evaluation of capsular and vascular invasion [6]. The reported frequency of follicular carcinoma in these nodules has decreased over time due to refinements in histologic criteria [21]. Although diagnostic lobectomy has traditionally been recommended, up to 65–70% of these lesions are benign and when malignant, they usually are low-risk malignancies such as the follicular variant of papillary carcinoma or NIFTP [4]. This results in a high rate of potentially avoidable surgeries and associated complications, including hypoparathyroidism and recurrent laryngeal nerve injury. Molecular testing has improved risk stratification in indeterminate nodules and reduced unnecessary surgery due to its high negative predictive value [7]. Nonetheless, its routine use is constrained by cost and limited access in low- and middle-income countries [22, 23, 19]. 

In the absence of molecular testing, integrating structured ultrasound systems (e.g., TI-RADS) with Bethesda classification has emerged as a relevant strategy to identify low-risk candidates for AS. Hong et al. [24] proposed a scoring model combining cytology and K-TI-RADS, defining four risk groups: very low (< 3%), low (3–30%), high (30–90%), and very high (> 90%), with AS considered suitable for low- and intermediate-risk groups. Lee et al. [25] observed that suspicious ultrasound features increased malignancy risk in Bethesda III nodules. Hirokawa et al., [26] found that combining cytology, suspicious ultrasound features, size ≥ 30 mm, and a volumetric doubling rate ≥ 1/year helped identify nodules amenable to follow-up. These findings support multimodal risk stratification as a cost-effective approach for managing Bethesda IV nodules with low malignancy potential [15, 17, 27]. Evidence from Bethesda V–VI nodules also suggests feasibility of AS: studies from Asia, North America, Europe, and Latin America [12–14, 18, 28–30] report low growth rates, indicating that surgery may be deferred in select cases, potentially reducing both complication rates and treatment costs (e.g., molecular testing or lifelong hormone therapy).

This study reports the largest published cohort of Bethesda IV nodules managed with AS to date, updating a prior series [15] with extended follow-up (71% of patients > 24 months). Among nodules < 4 cm and TI-RADS 2–4, 19.6% showed significant growth, 14.3% underwent surgery, and malignancy was confirmed in 60% of operated cases. These findings align with previous reports [16, 17, 26, 27]. Additionally, the 48-month stability rate approached 87%, reinforcing the feasibility of this strategy in well-selected patients.

Malignancy rates were comparable between nodules resected for growth and those removed despite stability (33% vs. 30%), suggesting that size increase alone may not be a reliable predictor. With strict selection criteria, AS remains a clinically viable option for Bethesda IV nodules, consistent with global experience. Decision models from the U.S. indicate that surgery, molecular testing, and observation yield similar quality-adjusted life years (QALYs), with minimal differences across strategies [31]. 

This study found a 7% overall malignancy rate, mostly comprising low-risk PTC. As in all AS cohorts, the lack of histological confirmation in non-operated patients is a key limitation, since benignity or malignancy cannot be definitively established without surgery. Accordingly, malignancy rates vary by denominator: 33% among growing nodules removed surgically, 46% among all operated patients, and 7% for the full cohort. The latest Bethesda classification [32] similarly highlights the difficulty of lacking surgical confirmation in many patients. Among those diagnosed with malignancy, most had low-risk PTC, unlikely to impact prognosis. The predominance of conventional PTC among malignant cases is consistent with previously published surgical series of Bethesda IV nodules, as some PTCs may present with a predominantly follicular architectural pattern on cytology with incomplete nuclear features, not meeting the threshold for a Bethesda V or VI classification. Furthermore, the exclusion of TI-RADS 5 nodules at enrollment reduced the likelihood of including nodules with overtly suspicious imaging characteristics.

These results reinforce the feasibility of AS in selected cases while admitting its inherent limitations. Adherence to monitoring protocols remains essential to reduce the risks of this approach.

The multivariable analysis did not identify significant predictors of growth, suggesting multifactorial influences beyond conventional clinical and imaging features. However, initial nodule size was independently associated with surgery, possibly reflecting clinician preference to operate on larger nodules regardless of documented progression.

Radiologists routinely apply TI-RADS in thyroid nodule evaluation. Despite interobserver variability, final categorization shows acceptable agreement between experts and non-experts [33–35]. Similarly, Bethesda classification demonstrates substantial concordance between general and expert pathologists, especially for indeterminate categories [35–37]. Reflecting real-world conditions, our study did not include centralized review or extraction of individual descriptors, relying instead on standardized TI-RADS and Bethesda classifications used in clinical practice.

Although a ≥ 3 mm growth threshold falls within interobserver variability for ultrasound, consistent examiner and equipment use is rarely feasible in routine care. Decisions are thus made under conditions of measurement uncertainty. While this may affect risk stratification and lead to misclassification, it mirrors actual diagnostic workflows and strengthens generalizability beyond specialized centers.

Several limitations warrant consideration. This retrospective analysis of a prospectively designed cohort was influenced by surgeon judgment. Although the proposed follow-up schedule represented the intended protocol at enrollment, strict adherence cannot be guaranteed for all patients given the non-centralized, real-world conditions of this study.

No systematic registry was maintained for patients evaluated but not offered or who declined AS. AS was generally offered to all patients meeting the predefined inclusion criteria and the same surgical team participated throughout the study period. To provide partial context regarding patient selection, during 2021 to 2024, 2,494 patients underwent thyroidectomy at the participating institutions, of whom 192 had Bethesda IV cytology. During this same period, 81 patients with Bethesda IV nodules meeting inclusion criteria were enrolled in the AS program, suggesting that approximately 30% of eligible patients were managed non-operatively during this interval.

Unmeasured variables, such as comorbidities or patient preference may have introduced selection bias. Nonetheless, all patients were initially evaluated as candidates for diagnostic thyroidectomy. In the absence of individualized clinical judgment, they would have routinely undergone surgery. While this introduces potential bias, it may also reflect a favorable subset for whom AS is a reasonable and personalized strategy. The absence of a contemporaneous control group, such as patients with Bethesda IV nodules who underwent immediate surgery, limits direct comparison between AS and surgical management.

It should be acknowledged that the standard definition of significant nodule growth requires a 20% increase in two dimensions (of at least 2 mm each); however, as growth was assessed in a single dimension in our clinical protocol, this two-dimension criterion could not be applied retrospectively.

The present study was conceived as a necessary preliminary step toward a more rigorous prospective design. At the time this cohort was initiated, evidence on AS for Bethesda IV nodules was limited, making it methodologically appropriate to first characterize the real-world behavior of this intervention and determine whether its outcomes were sufficiently promising to justify a future randomized trial, while explicitly acknowledging the risk of bias inherent to its retrospective nature.

Limited follow-up is a key limitation, as it may miss late-onset malignancies or delayed progression. Still, the absence of significant growth suggests immediate surgery may be unnecessary in selected patients, particularly where molecular testing is unavailable. Notably, a substantial subset had over two years of follow-up, supporting nodule stability and the feasibility of AS in real-world practice.

## Conclusion

In this cohort of Bethesda IV nodules, AS showed stability at 48 months, a low growth rate, and a low overall malignancy risk, with low-risk PTC representing most cases. These findings indicate that the use of protocols based on clinical and US criteria, can reduce avoidable diagnostic surgeries while maximizing resources in locations with limited access to molecular test. While acknowledging the limitations of the design and diagnostic heterogeneity, the findings reflect real-world performance and support multicenter prospective studies with specific selection criteria and long-term follow-up.

## Supplementary Information

Below is the link to the electronic supplementary material.

Supplementary Material 1

## Data Availability

The data that support the findings of this study are available from the corresponding author upon reasonable request.
