# Supplementary material for: A real-world experience of active surveillance in Bethesda IV thyroid nodules
Source: Endocrine. 2026 Jul 13;91(1):233. doi: 10.1007/s12020-026-04678-5 (PMC13364893; doi:10.1007/s12020-026-04678-5)

**Supplementary file.**

**Supplementary table 1. Outcomes of patients who underwent surgery**

| Variable | **Group operated with growing nodules (n=17)** | **Group operated with stable nodules (n=9)** |
| --- | --- | --- |
| **Histology** |  |  |
| Benign | 8 | 5 |
| Papillary carcinoma | 6 | 2 |
| Minimally invasive follicular carcinoma | 2 | 1 |
| Medullary carcinoma | 1 | 0 |
| No data | 0 | 1 |
| **T stage for malignant tumors** |  |  |
| T1 | 5 | 3 |
| T2 | 3 | 0 |
| T3 | 1 | 0 |
| **N stage for malignant tumors** |  |  |
| N1 | 0 | 0 |
| ATA risk of recurrence |  |  |
| **Low risk tumors** | 13 | 9 |
| **Intermediate risk tumors** | 4 | 0 |
| **Type of surgery** |  |  |
| Lobectomy, n | 11 | 7 |
| Total thyroidectomy, n | 5 | 2 |
| Completion thyroidectomy, n | 1 | 0 |
| **Surgical complications** | |  |
| Definitive hypoparathyroidism | 1 | 0 |
| Unilateral RLN paralysis | 0 | 1 |
| **Time to surgery (months)** | 31.8±20.4 (2-67) | 36.1±20.5 (14-77) |
| **RAI ablation** | 4 | 0 |

**Supplementary table 2. Characteristics of nodules according to nodule size**

|  | **0-9 mm** | **10-19 mm** | **20-40 mm** | **Total** | **P value** |
| --- | --- | --- | --- | --- | --- |
| **n** | 25 | 113 | 46 | 184 |  |
| **Size at initial evaluation (mm)** |  |  |  |  |  |
| Mean size +- SD (range) | 7,7±1,3 | 14,1±2,8 | 25,8±5,1 |  | 0.00 |
| TI-RADS 2 | 1 | 7 | 2 | 10 | 0.09 |
| TI-RADS 3 | 7 | 37 | 25 | 69 |  |
| TI-RADS 4 | 17 | 69 | 19 | 105 |  |
| **Size change (mm)** | 1,1±2,3 (-3 to 7) | 0,8±4,1 (-10 to 13) | -0,3±8,2 (-28 to 22) |  |  |
| **(**Mean +- SD (range) |  |  |  |  |  |
| **Time of follow-up (**Mean +- SD) | 35,7±26,2 | 50,3±30,4 | 42,3±29,9 |  | 0.05 |
| **>24 m (%)** | 16 (64%) | 84 (74%) | 32 (69%) | 132 (71.7% | 0.6 |
| **Growth>3 mm (n, %)** | 3 (12%) | 23 (20,3%) | 10 (21,7%) | 36 (19,6%) | 0.57 |
| **Growth >20% initial diameter** | 8 (32%) | 25 (22.1%) | 10 (21.7%) | 43 (23.4%) |  |
| **Underwent surgery (n, %)** | 2 (8%) | 14 (12,4%) | 10 (21,7%) | 26 (14.3%) | 0.19 |
| **Malignancy in operated** | 2 (100%) | 6 (42,8%) | 4 (40%) | 12 (46,1%) | 0.28 |
| **Malignancy in growth> 3mm** | 1 (33%) | 5(21,7%) | 3 (33,3%) | 12(33%) | 0.82 |
| **Malignancy by group** | 2 (8%) | 6 (5,3%) | 4 (8,7%) | 12 (46%) | 0.69 |
| **Nodules without growth> 3 mm at 48 m** | 93,90% | 89,90% | 82,30% | 88,60% | 0.70 |
| **Nodules without surgery at 48 m** | 93,90% | 93,20% | 82,30% | 90,60% | 0.11 |

**Supplementary table 3. Comparison between growth criteria by TIRADS (A) and tumor size (B)**

| **Growth> 20% initial diameter** | **TI-RADS 2** | **TI-RADS 3** | **TI-RADS 4** | **Total** |
| --- | --- | --- | --- | --- |
| **No** | **8 (80%)** | **56 (81.2)** | **77 (73.3%)** | **141 (76.6%)** |
| **Yes** | **2 (20%)** | **13 (18.8%)** | **28 (26.75)** | **43 (23.4%)** |

| **Growth> 20% initial diameter** | **0-9 mm** | **10-19 mm** | **20-40 mm** | **Total** |
| --- | --- | --- | --- | --- |
| **No** | **17 (68%)** | **88 (77.9%)** | **36 (78.3%)** | **141 (76.6%)** |
| **Yes** | **8 (32%)** | **25 (22.1%9** | **10 (21.75)** | **43 (23.4%)** |

**Supplementary figure 1. Behavior of Bethesda IV nodules according to size.**

**Supplementary figure 2.**

**Evolution of thyroid nodules with Bethesda IV cytology under AS, showing the distribution according to nodule size, and the histopathological results in cases undergoing surgery.**


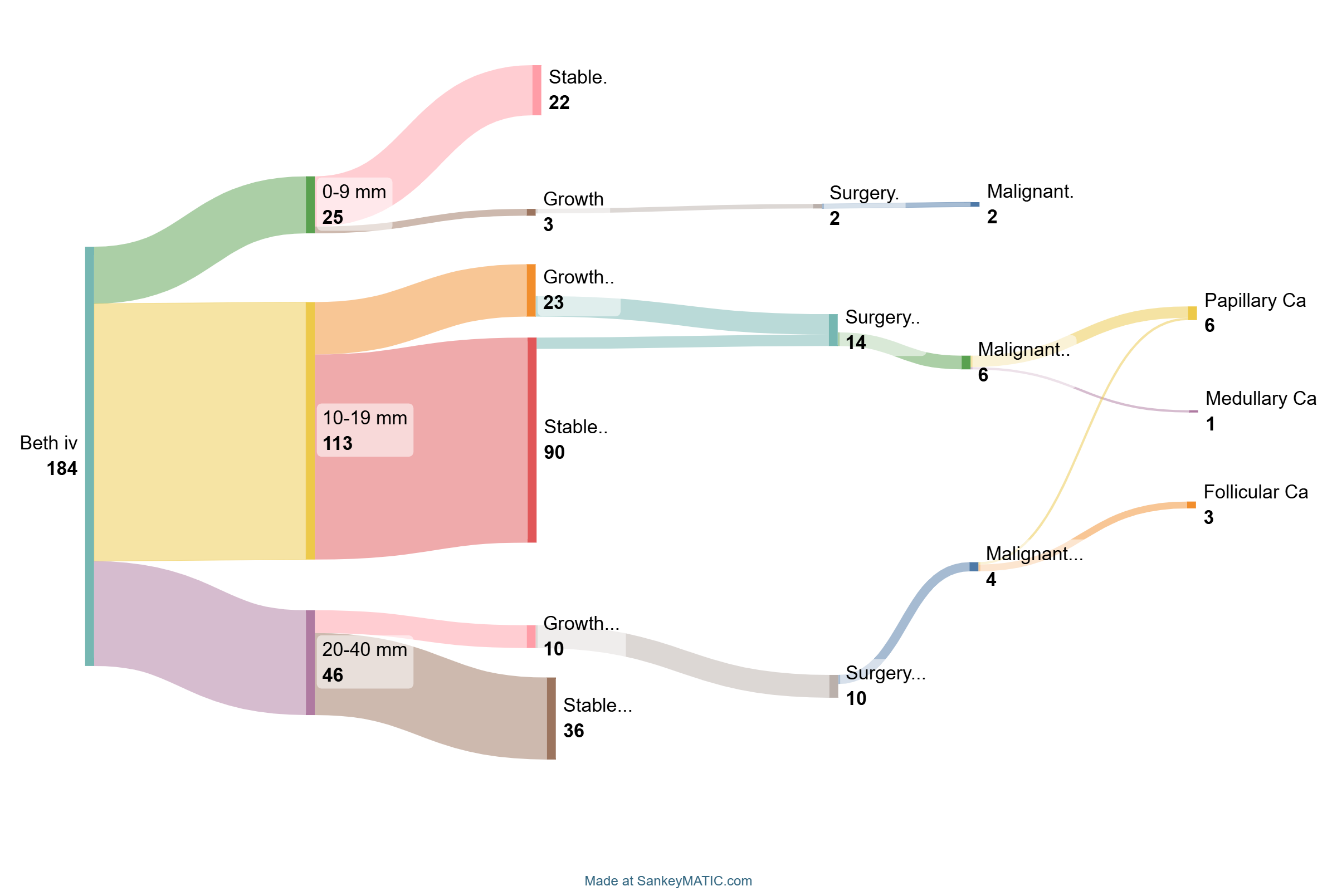

Supplement: Supplementary file 1 — Supplementary Material 1 [file 12020_2026_4678_MOESM1_ESM.docx]
